# Plasma Albumin Induces Cytosolic Calcium Oscilations and DNA Synthesis in Human Cultured Astrocytes

**DOI:** 10.1155/2014/539140

**Published:** 2014-05-22

**Authors:** Lorena Vega-Zelaya, Guillermo J. Ortega, Rafael G. Sola, Jesús Pastor

**Affiliations:** ^1^Clinical Neurophysiology, The Epilepsy Unit, University Hospital La Princesa, Calle de Diego León 62, 28006 Madrid, Spain; ^2^Neurosurgery, The Epilepsy Unit, University Hospital La Princesa, 28006 Madrid, Spain

## Abstract

So far, a little is known about transition from normal to focal epileptic brain, although disruption in blood-brain barrier and albumin had recently involved. The main objective of this work is to characterize the response of cultured human astrocytes to plasma albumin, including induction of DNA synthesis. Cortical tissue was obtained from 9 patients operated from temporal lobe epilepsy. Astrocytes were cultured for 3-4 weeks and cytosolic calcium concentration ([Ca^2+^]_*c*_) was measured. Bovine and human plasma albumin were used. We observed that low albumin concentration decreases [Ca^2+^]_*c*_, while higher concentration, induces increase in [Ca^2+^]_*c*_. It was shown that increase in [Ca^2+^]_*c*_ was mediated by inositol 1,4,5-trisphosphate and released from internal stores. Increase in [Ca^2+^]_*c*_ was reduced to 19% by blocking the transforming growth factor-beta (TGF-**β**R) receptor. Albumin induces DNA synthesis in a dose-response manner. Finally, induction of DNA synthesis can be partially blocked by heparin and block of TGF-**β**; however, the combination of both incompletely inhibits DNA synthesis. Therefore, results suggest that mechanisms other than Ca^2+^ signals and TGF-**β** receptor activation might induce DNA synthesis in a lesser degree. These results may be important to further understand the mechanisms involved in the transition from normal to focal epileptic brain.

## 1. Introduction

Epilepsy is a major neurological disorder, affecting about 0.5–2% of the global population and approximately 20–30% of these patients are resistant to drug therapy [1–3]. Despite advances in the understanding of epilepsy, the pathophysiological bases of the epileptogenesis in humans are unclear, but it is well known that blood-brain barrier (BBB) disruption is associated with epilepsy [4–7].

Recently, it has been shown that astrocytes may be involved in epileptogenesis in rats and also in humans [8–10]. And most importantly, serum albumin (s-Alb) has been proposed as a potent epileptogenic factor, probably acting through the transforming growth factor beta receptor (TGF-*β*R) [11]. This effect is mediated through endocytosis of albumin (Alb) and could be the trigger for changes in DNA expression, reducing the potassium inward-rectifier conductance Kir. This modification could be responsible for increase in extracellular K^+^ concentration and hyperexcitability [12].

Albumin (C_123_H_193_N_35_O_37_) is the most abundant protein in blood, representing more than 50% of total protein. Its molecular weight is 2754.06 g/mol and its concentration in plasma is around 35–55 mg/mL [13, 14]. Among its functions is the binding of several polar lipids, especially lysophosphatidic acid (LPA), other fatty-acids, and sphingomyelins derived from platelet activation [15], and is the main component in maintaining the osmotic plasma pressure.

It is well known that Alb obtained after coagulation, serum-albumin (s-Alb), could activate several types of cells, including xenopus oocytes, PC12, and fibroblasts microglia [7, 16–19]. Moreover, pure Alb can activate microglial cells [20]. However, it was also demonstrated that plasma albumin (p-Alb) obtained from plasma without coagulation was able to induce a dual response on the cytosolic calcium concentration ([Ca^2+^]_*c*_) in rat astrocytes in culture and slices [21]. Indeed, low p-Alb concentrations reduced the [Ca^2+^]_*c*_ through an Alb receptor, while higher p-Alb concentrations increased the [Ca^2+^]_*c*_ in a dose-dependent manner, eliciting calcium-waves that travelled by gap-junctions, probably involving inositol 1,4,5-trisphosphate (IP_3_, [22]). These calcium oscillations and waves were associated with DNA synthesis [21].

So far, none of both models of astrocyte's activation have been demonstrated in human tissue, but only in rat astrocytes [11, 21]. The main goal of this work is to evaluate the response of cultured human astrocytes to p-Alb, obtained from adult human beings operated from drug-resistant epilepsy. We show that p-Alb elicits [Ca^2+^]_*c*_ signals and new DNA synthesis by a mechanism similar to that described previously [21, 22] in rat astrocytes. Therefore albumin works as a signaling molecule in human astrocytes and may be important to understand how brain epileptic focus can be generated.

Preliminary results were published as an abstract form [10].

## 2. Material and Methods

### 2.1. Patients

Human brain tissue was obtained from patients diagnosed of temporal lobe epilepsy (TLE) and operated to control their seizures. The patient's consent was obtained in all cases according to the Declaration of Helsinki and all protocols were approved by the Institutional Ethical Committee (Hospital de la Princesa, Madrid, Spain). A mass of brain tissue (approximately 3 g) containing grey and white mater, obtained from a nonspiking region of temporal lateral cortex, was obtained from 9 patients (4 males and 5 women) suffering from drug-resistant temporal lobe epilepsy, all with a well-documented medical history.

Surgical treatment of the epileptic patients required the exact localization of the epileptic focus. Patients were presurgically evaluated according to La Princesa's protocol, published elsewhere [23, 24]. Briefly, all patients were studied with scalp electroencephalography (EEG), interictal single photon emission computer tomography (SPECT) with ^99m^Tc-HmPAO, magnetic resonance imaging (MRI) 1.5 T, and video-electroencephalography (v-EEG) using 19 scalp electrodes according to the international 10–20 system. In some patients, six-contact platinum foramen ovale electrodes (FO), with 1 cm center-to-center spacing (AD-Tech, Racine, USA), were inserted bilaterally under general anesthesia, as previously published [25]. During the operation, resection was guided by electrocorticography (ECoG) placing a 4 × 5 electrode grid (lateral neocortex) and a 1 × 8 electrode strip (uncus and parahippocampal gyrus) directly over the cortex.

### 2.2. Culture of Astrocytes

Human astrocytes were processed according to the protocol described by Vreugdenhil et al. [26], after minor modifications described below. The piece was introduced in a sterilized solution at 4°C, containing (in 10^−3 ^mol/L) 120 NaCl, KCl 5, CaCl_2_ 1, MgCl_2_ 2, 10 HEPES, and D-glucose 25, pH 7, bubbled with 100% O_2_. The tissue was enzymatically dissociated for 1 hour at 32°C with constant oxygenation and agitation and after that the tissue was mechanically dissociated and cultured in Neurobasal A medium containing 10% fetal bovine serum (FBS) (Sigma-Aldrich, Madrid, Spain) at 10^5^ cells/mL in glass coverslip (*ϕ* = 13 mm) coated with poly-L-lysine. The culture medium was replaced by fresh one every 4 days. Cultures were used for experiments at 20–27 days.

Anti-body against glial fibrillary acidic protein (GFAP) (Sigma-Aldrich. Madrid, Spain), diluted at 1 : 100 was used onto fixed cells [27] to identify astrocytes and anti-NeuN (Chemicon International. Madrid, Spain), diluted at 1 : 100, was used to identify neurons.

### 2.3. Measurement of Cytosolic Calcium Concentration

Astrocytes were loaded with the fluorescent dye Fura-2 AM (10^−6 ^mol/L) and probenecid (1 (mol/L)/L) (Sigma-Aldrich, Madrid, Spain) and pluronic acid (0.05%) (Molecular Probes, Barcelona, Spain) dissolved in Krebs-HEPES solution (pH 7.4), containing (in 10^−3 ^mol/L) NaCl 145, KCl 5.9, MgCl_2_ 1.2, CaCl_2_ 2.5, HEPES 10, and glucose 10 during 45 minutes at 37°C. Several rinses with Krebs-HEPES were then performed.

Cytosolic calcium concentration ([Ca^2+^]_*c*_) was measured at room temperature from several regions of interest (ROI [28]). Cells were alternatively illuminated at 340 and 380 nm and the emitted light at 520 nm was collected by a photomultiplier and analyzed with the software Cell-R (Olympus, Madrid, Spain).

Results were expressed as ratio F340/F380 (*R*) and basal values (*R*_0_) were subtracted (Δ*R* = *R* − *R*_0_ [29]).

### 2.4. Assay of DNA Synthesis

Astrocytes grown to 50% confluence on poly-L-lysine coated coverslips were exposed for 24 hr to serum-free medium to inhibit cell division, followed by 24 hr in serum-free test medium with 10^-5 ^mol/L bromodeoxyuridine (BrdU) (Sigma-Aldrich, Madrid, Spain) [21, 30]. Cells were washed with phosphate buffered saline (PBS) and fixed with paraformaldehyde 3.7% during 20 min at room temperature, treated with 2 mol/L HCl at 37°C for 20 min to denature DNA, neutralized with 0.1 mol/L borax, and permeabilized with 1% Triton X-100. BrdU was labeled with monoclonal antibody mouse anti-BrdU (Sigma-Aldrich, Madrid, Spain), during 2 hours (1 : 40 dilution in PBS with 2% fetal bovine serum). Cells were washed again in PBS and incubated during 45 minutes with fluorescence secondary antibody, goat anti-mouse (1 : 400 dilution in PBS with 2% fetal bovine serum) (Molecular Probes, Barcelona, Spain). All nuclei were labeled with 4′,6-diamino-2-phenylindole (1 *μ*g/mL) (Molecular Probes, Barcelona, Spain) and BrdU-positive nuclei were expressed as a percentage of total. More than 200 nuclei were counted per coverslip. Samples were examined by blinded counting, the representative fields were taken randomly, and the final value is the mean of three different experiments.

### 2.5. Plasma Albumin from Patients

In a small number of patients (*n* = 3) plasma albumin was obtained from blood during the operation. A sample of venous blood (3 mL) was obtained in presence of 10^−3 ^mol/L EDTA (Invitrogen, Barcelona, Spain) to avoid clotting. Plasma albumin concentration was determined in a different sample obtained at the same time. The samples were centrifugated and cells discarded. The plasma supernatant was frozen at −74°C until the experiment was performed. At that moment, plasma samples were diluted according to the real plasma concentration measured.

### 2.6. Statistical Analysis

Statistical comparisons between groups were performed using either the Student's *t*-test or one way ANOVA for normal (Gaussian-fitted) data. The Mann-Whitney *U* test (sometimes rank-sum test) was used if normality failed. Paired Student's *t*-test was used when needed for paired data. Statistical analyses were performed with the commercial software SigmaStat 3.5 (Point Richmond, USA). Significance level was set at *P* < 0.05. Results are shown as means ± SEM, except otherwise indicated.

## 3. Results

Cells grew as a monolayer, and primary cultures reached the confluence between the third and fourth week.

Immunolabeling for astrocytes and neurons showed a virtual absence of neurons (<5%). Protoplasmic and fibrous astrocytes were morphologically identified.

### 3.1. Plasma Albumin Elicits [*Ca* ^2+^]_*c*_ in Astrocytes

**Figure 1:**
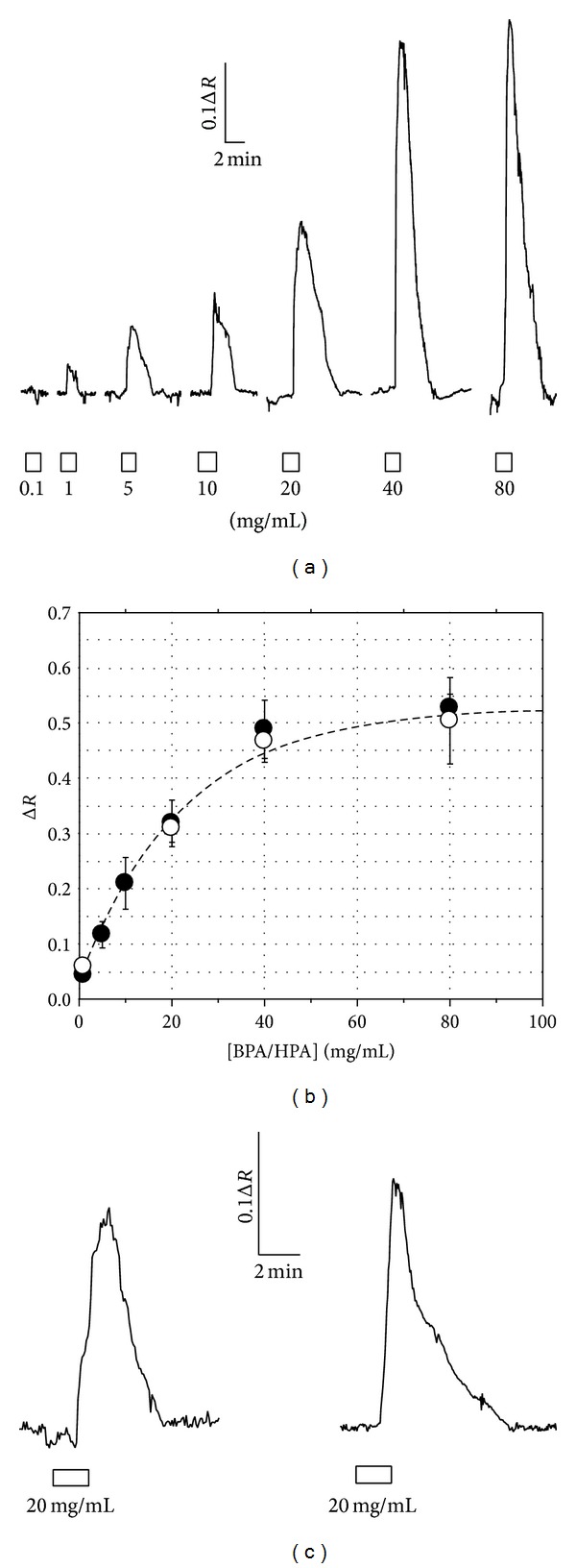
Human astrocytes respond to albumin increasing cytosolic calcium concentration. (a) Effects of BPA onto [Ca^2+^]_*c*_ at different concentrations from 0.1 to 80 mg/mL. (b) Mean height of [Ca^2+^]_*c*_ in response to increasing BPA concentrations (solid dots, *n* = 72 cells obtained from 8 different patients). Data are fitted (solid line) to the function*f*(BPA) = −0.07 + 0.55(1 − *e* ^−BPA/20.49^); *r* = 0.990. Solid arrowhead shows the EC_50_ at 14.5 mg/mL. Empty dots represent [Ca^2+^]_*c*_ responses to HPA (*n* = 23 cells from 4 patients). Data are fitted (dashed line) to the function (HPA) = 0.03 + 0.49(1 − *e* ^−HPA/22.22^); *r* = 0.979. Empty arrowhead shows the EC_50_ at 13.8 mg/mL. (c) A similar effect onto [Ca^2+^]_*c*_ was observed using either BPA (left) or human plasma albumin (HPA) obtained from the patient (right) at the same concentration (20 mg/mL). Lower empty bars show time of albumin application (in min). Concentrations are given in mg/mL.

It has been shown that bovine plasma albumin (BPA) induces oscillations in the [Ca^2+^]_*c*_ in cortical rat astrocytes in a dose-dependent manner [21]. In Figures 1(a) and 1(b) it is shown that human astrocytes obtained from adults also respond in double mode to BPA. In fact, [Ca^2+^]_*c*_ decreased to low dose of BPA (0.1 mg/mL) but the maximum amplitude for [Ca^2+^]_*c*_ increased with BPA concentration between 1 mg/mL and 80 mg/mL.

The same effect was observed using human p-Alb (HPA) at the same concentration, obtained from the patient instead of BPA (Figures 1(b) and 1(c)). Taking into account that no differences were observed between HPA and BPA, in the following, we will always use the last one.

**Figure 2:**
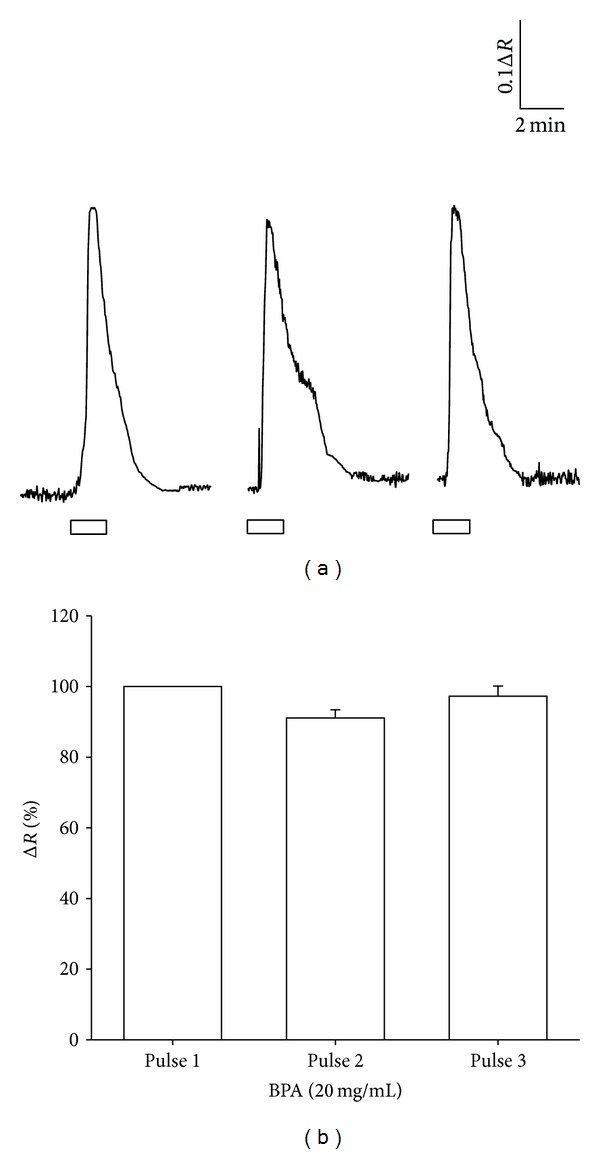
Albumin cannot induce adaptation. (a) Consecutive responses to 20 mg/mL of BPA 5 min apart. Lower empty bars show time of BPA application (in min). Concentrations are given in mg/mL. (b) Bar diagram shows absence of adaptation (*n* = 32 cells from 5 patients).

Consecutive applications of BPA failed to induce any significant decrease in the maximum amplitude in [Ca^2+^]_*c*_, as we can observe from Figure 2. This result shows that no adaptation in the response is induced by BPA.

Taken together, these results show that plasma albumin obtained from patients, such as commercially available BPA, induced modifications in [Ca^2+^]_*c*_, decreasing at low BPA concentration and increasing at higher concentrations. These responses do not show adaptation.

### 3.2. Plasma Albumin Releases Ca^2+^ from Internal Stores

It has been previously described that cytosolic calcium increase in astrocytes depends on calcium release from internal stores [21, 31]. We have studied this mechanism in our model.

**Figure 3:**
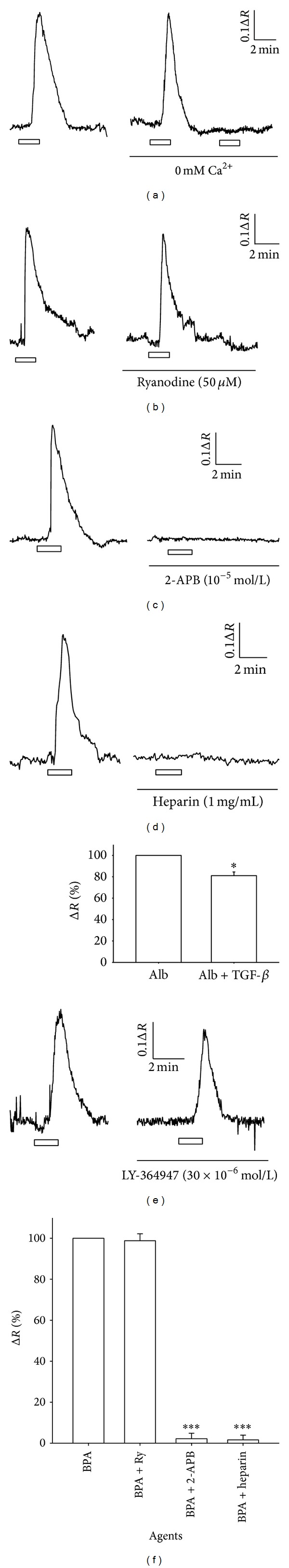
Cellular mechanisms involved in cytosolic calcium change. (a) Response to BPA (20 mg/mL) in 0-calcium (0 Ca^2+^ + 10^−3 ^mol/L EGTA). The first pulse of BPA can induce an increase in [Ca^2+^]_*c*_, but not the second, performed with presumably intracellular empty calcium-stores. (b) Response to BPA in presence of 50 × 10^−6 ^mol/L ryanodine. (c) Absence of response to BPA (20 mg/mL) in presence of 10^−5 ^mol/L 2-APB. (d) BPA fails to induce [Ca^2+^]_*c*_ increases in presence of heparin (1 mg/mL). (e) Response to BPA in presence of LY-364947 (30 × 10^−6 ^mol/L) showed a slight reduction in the maximum height of calcium increase (inset); *P < 0.05 for paired Student's *t*-test (*n* = 42 cells obtained from 3 patients). (f) Diagram showing the effects of several agents acting onto receptor/second-messenger systems (*n* = 53 cells, obtained from 8 patients); ***P < 0.001 for Student's *t*-test. Empty bars show application time of BPA (20 mg/mL) and solid bars show application of different agents (in min).

In fact, as we can observe from Figure 3(a), 20 mg/mL of BPA induced a [Ca^2+^]_*c*_ increase even in a calcium free-medium containing 0 Ca^2+^ + 10^−3 ^mol/L EGTA. However, a second trial failed to induce any change in [Ca^2+^]_*c*_, likely because the internal stores were emptied by the previous pulse.

We then addressed the second messengers involved. As it is shown in Figure 3(b) ryanodine 50 × 10^−6 ^mol/L (a concentration able to inactivate the calcium induce-calcium release mechanism) (Sigma-Aldrich, Madrid, Spain) did not modify the maximum peak of cytosolic calcium concentration (see also Figure 3(f)), but the 2-amino-etoxi diphenyl borate (2-APB) 10^−5 ^mol/L, a selective antagonist of the IP_3_ receptor, completely abolished the effect of BPA (Figures 3(c) and 3(f)). Furthermore, heparin (Mayne Pharma, Madrid, Spain), a different selective antagonist of the IP_3_ receptor, when intracellularly applied [32] abolished the response to BPA (Figures 3(d) and 3(f)). We did not observe any difference in the blocking degree induced by heparin and 2-APB.

The TGF-*β* receptor has been described to have a role in albumin-elicited DNA synthesis in rat astrocytes. To evaluate whether this receptor is somehow involved in the [Ca^2+^]_*c*_ movement described here, the calcium response to BPA was also evaluated in presence of an TGF-*β* type I receptor kinase inhibitor (LY-364947) (Calbiochem, Beeston, UK) at a concentration high enough to block the receptor (30 × 10^−6 ^mol/L, [33, 34]). As we can observe from Figure 3(e), the maximum height of [Ca^2+^]_*c*_, obtained in response to BPA (20 mg/mL), decreased a 19.0% (*P* < 0.05 for paired Student's *t*-test, *n* = 42 from 6 patients).

These results suggest that increases in [Ca^2+^]_*c*_ in human astrocytes are due to release from internal stores and are mediated through IP_3_ as second messenger, but not by calcium-induced-calcium release. Probably, heparin acts extracellularly, blocking the interaction between Alb and its receptor. The rise in [Ca^2+^]_*c*_, nevertheless, depended in a little degree (<20%) on TGF-*β* receptor. Therefore, we can rule out that the main effect of BPA onto [Ca^2+^]_*c*_ is through the TGF-*β* receptor.

### 3.3. Viability of Astrocytes after Long-Duration Incubation in Heparin

**Figure 4:**
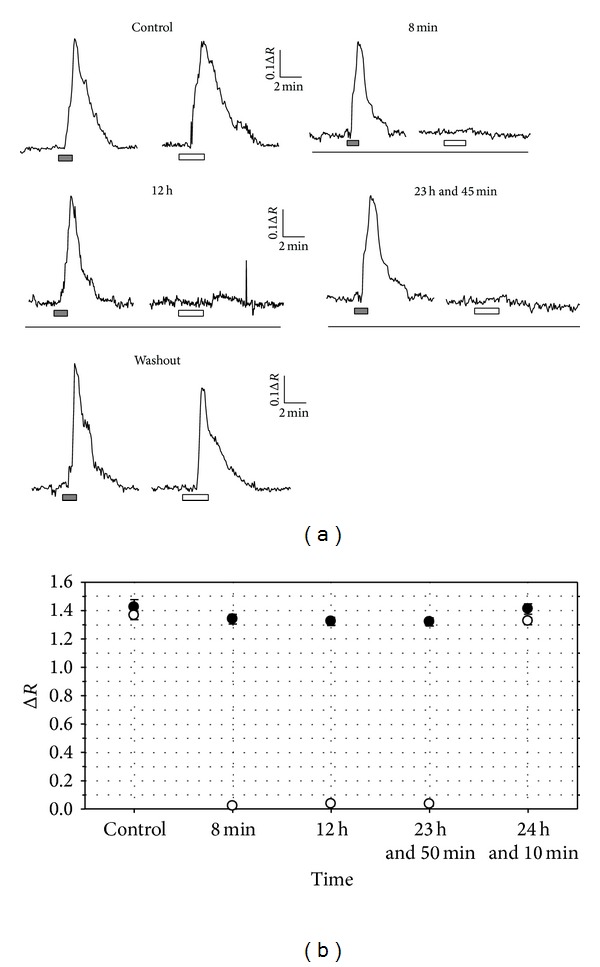
Functionality of astrocytes after long-duration incubation in heparin. (a) Perfusion of glutamate (5 × 10^−4 ^mol/L, grey bars) and BPA (20 mg/mL, empty bars) in control induced [Ca^2+^]_*c*_ increase. Then, we perfused heparin (1 mg/mL) during 24 hours. Eight minutes after heparin, glutamate was able to induce a response similar to the control one, but albumin failed to modify the [Ca^2+^]_*c*_. At 12 hours and at 23 hours and 45 minutes in heparin, we obtained the same result. Nevertheless, at 24 hours and 10 minutes, that is, 10 minutes after having washed out heparin, both agents induced a [Ca^2+^]_*c*_ increase similar to the control. Time of heparin indicated at the top. Grey bars show application of glutamate and empty bars show application of BPA. Solid bars show presence of heparin. (b) Diagram showing the maximum height of [Ca^2+^]_*c*_ increase induced by glutamate (solid dots) and BPA (empty dots).

Before addressing the effect of heparin and albumin onto DNA synthesis, we need to demonstrate the functionality of astrocytes to long exposures of heparin. Coverslips were placed onto the recording chamber, astrocytes were loaded with Fura-2 AM, and we consecutively applied glutamate (5 × 10^−4 ^mol/L) and albumin (20 mg/mL). Both glutamate and Alb are able to release calcium from internal stores. As we can observe from Figure 4(a) (control), both glutamate and albumin induced [Ca^2+^]_*c*_ increase. Then, we perfused heparin (1 mg/mL) during 24 hours. Eight minutes after heparin, glutamate was able to induce a response similar to the control one, but albumin failed to modify the [Ca^2+^]_*c*_. At 12 hours and 23 hours and 45 minutes in heparin, we obtained the same result. Nevertheless, at 24 hours and 10 minutes, that is, 10 minutes after having washed out heparin, both agents induced a [Ca^2+^]_*c*_ increase similar to the control (Figures 4(a) and 4(b)).

This result denotes that heparin can block cytosolic calcium oscillations induced by plasma albumin during long periods without affecting the cell viability.

### 3.4. Albumin Elicits DNA Synthesis

Plasma albumin was demonstrated as a potent agent inducing DNA synthesis in rat astrocytes [21]. This is a very important step in the theoretical framework of astrocyte-mediated epileptogenesis, because a plausible link between albumin activation of astrocytes and changes in neuronal excitability could be through modifications in gene-expression. Additionally, some of these changes have been really observed in a different model of rat astrocytes [11].

**Figure 5:**
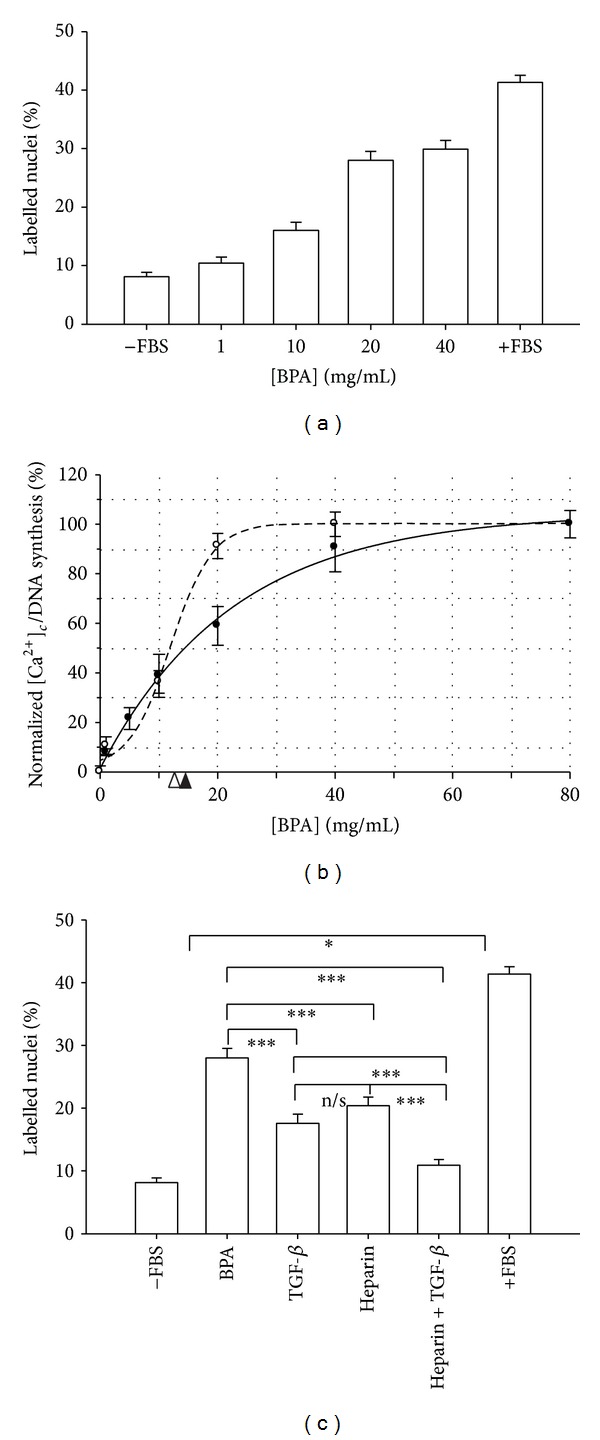
DNA synthesis induced by plasma albumin. (a) Effect of different BPA concentrations onto DNA synthesis. (b) Comparison of normalized [Ca^2+^]_*c*_ (empty dots) and DNA synthesis (solid dots) induced by BPA. The [Ca^2+^]_*c*_ was fitted to an exponential function (*f*(PBA) = 103.9(1 − *e* ^−BPA/21.74^); *r* = 0.994) and DNA synthesis to an sigmoid function (*f*(BPA) = 0.08 + 100.5/(1 + *e* ^−(BPA−6.0)/1.83^); *r* = 0.998) by the least-square method. Solid arrowhead indicates the EC_50_ for [Ca^2+^]_*c*_ increases (14.5 mg/mL) and solid arrow marks the EC_50_ for DNA synthesis (10.3 mg/mL). (c) DNA synthesis is partially blocked by LY-364947 (30 *μ*M) and heparin (1 mg/mL). Combination of heparin + LY-364947 reduces in a higher degree DNA synthesis; however, it keeps some amount of DNA synthesis induced by plasma albumin. *P < 0.05, **P < 0.01, and ***P < 0.001 for Student's *t*-test.

When astrocytes were cultured in presence of increasing concentrations of BPA (1, 10, 20, and 40 mg/mL), a dose-response relationship between DNA synthesis and BPA concentration was observed (Figure 5(a)).

We assessed the relationship between increases in [Ca^2+^]_*c*_ and DNA synthesis induced by BPA. As we can observe from Figure 5(b), the relationships between [Ca^2+^]_*c*_ and DNA synthesis with BPA are rather different. In fact, the half maximal effective concentration (EC_50_) of albumin for [Ca^2+^]_*c*_ is 14.5 mg/mL and 10.3 mg/mL for DNA synthesis. Furthermore, both curves are clearly different.

We have shown that BPA can induce increase in DNA synthesis but what is the mechanism? To address this question, astrocytes were incubated in presence of 20 mg/mL BPA and in either heparin (1 mg/mL) or LY-364947 (30 *μ*M).

From Figure 5(c) we can observe that blocking of [Ca^2+^]_*c*_ increase by heparin can reduce the DNA synthesis about a 40% compared with BPA. However, the percentage of labeled nuclei was still significant with respect to control (*P* < 0.001, ANOVA). A similar result was obtained using LY-364947 (30 × 10^−6 ^mol/L) in presence of BPA; the increase in DNA synthesis was reduced approximately to a 50%, in the same range than in the reduction induced by heparin. There was not any difference between heparin and TGF-*β* receptor antagonist groups (*P* > 0.05, Mann-Whitney *U* test).

So, both mechanisms are responsible approximately for the same amount of DNA synthesis induced by 20 mg/mL BPA, but is there any other mechanisms? To answer this question, astrocytes were incubated with BPA (20 mg/mL) + heparin (1 mg/mL) + LY-364947 (30 *μ*M). However, the percentage of labeled nuclei was still different than control (*P* < 0.05, ANOVA test). This result demonstrates that mechanisms other than TGF-*β* receptor and [Ca^2+^]_*c*_ increase are involved in DNA synthesis.

## 4. Discussion

In this work we show that p-Alb, from both humans as bovine, induces calcium responses in human astrocytes obtained from adult patients, in the same way as previously reported in newborn rat astrocytes [21, 22]. The similarities between both models include IP_3_ mediation, [Ca^2+^]_*c*_ decrease to low p-Alb concentrations, and DNA synthesis induction in a dose-dependent manner. But not only DNA synthesis can be induced through the IP_3_ pathway, as have been showed in rats [11], but also TGF-*β* receptor induces, in a similar degree, DNA synthesis without the involvement of changes in [Ca^2+^]_*c*_. We have also demonstrated that heparin inhibits increases in cytosolic calcium in a fast and reversible way.

So far, albumin has been shown to activate different types of cells, including rat astrocytes in culture [21, 22] and in brain slices [11]. Recently, astrocytes and albumin have been related to epilepsy [8], mainly through disruption of BBB. However, at the present, no reports have been published about activation of human astrocytes by albumin. So, it was be very important to demonstrate that this possibility and cultured astrocytes can be a suitable model [35]. In fact, it has been demonstrated that cultured astrocytes from human adults can express functional differential properties [36].

Plasma albumin, both obtained from commercial suppliers and from patients, prompted the same response as previously observed in rat astrocytes. In fact, we have observed a dual response, decreasing [Ca^2+^]_*c*_ at low doses and proportionally increasing at higher doses. Also, we have demonstrated that cytosolic calcium increase depends on the same mechanisms of second messenger, IP_3_ [22]. Therefore, is it not unreasonable to suppose that the fall in [Ca^2+^]_*c*_ depends directly on p-Alb, while [Ca^2+^]_*c*_ increases depend on the unknown phospholipid bound to albumin. In the same way, we can assume that calcium increase mediates synthesis of DNA, as we observed in the rat astrocytes [21].

A key-mechanism in epileptogenesis can be the induction of DNA synthesis in astrocytes. This effect has been demonstrated* in vitro* [11] and also in cultured rat astrocytes [21]. We have shown here that p-Alb can also induce DNA synthesis in human astrocytes obtained from adults. Although we have not showed changes in expression of genes, this is a real possibility demonstrated in rats [11]. This change in gene-expression could be responsible for changes in excitability, as have been demonstrated in rat, and might be the root of focal epileptogenesis in humans. However, much more work need to be done before demonstrating this hypothesis. In fact, TGF-*β* receptor shows upregulation in tissue resected from epileptic patients [37]. However, it is debated whether this upregulation is cause or effect for epilepsy.

Both models describing DNA synthesis so far, in rat and human, share important features [10, 11, 21]. Both of them need disruption in BBB before allowing albumin to go to the extracellular space and both of them can activate astrocytes and induce DNA synthesis after albumin exposure. Nevertheless, there are also significant differences. The type of albumin used is different, depending on the presence of coagulation, what implies differences in the pathophysiological process. Besides, kinetics is very different, because in one case albumin need be introduced into the cell and in the other a short exposure can induce cytosolic calcium increase and DNA synthesis. However, both mechanisms explain DNA synthesis by p-Alb in a similar degree. However, our results show that a third mechanism activated by p-Alb might induce DNA synthesis (Figure 5(c)). In fact, approximately a 10% of DNA synthesis cannot be blocked by a combination of heparin + TGF-*β* receptor antagonist.

We have found that heparin can block the rise in [Ca^2+^]_*c*_ in response to p-Alb. Taking into account its high molecular weight (1661.4 g/mol, C_36_H_60_O_55_S_9_) it seems unlikely that this effect would be due to an intracellular mechanism and, on other hand, there is no notice to authors about membrane mechanisms able to cross the membrane for a so big molecule. In fact, heparin acting in a selective way through IP_3_ receptors has to be introduced inside the cell [32]. Preliminary results suggest that heparin can specifically block the interaction among Alb and its putative membrane receptor. However, elucidating the exact mechanism of action for heparin was clearly out of the goal of this paper, and obviously more experiments are needed before establishing unequivocally this fact.

In summary, it would be extremely important to assess what of the mechanisms effectively applies in humans, in order to design pharmacological strategies. On the other hand, it is conceivable that more than one mechanism acts synergistically.

Any case, there is a real and exciting possibility to envisage a pathway running from the normal human brain to focal epilepsy after BBB disruption induced by a local insult [35]. This possibility opens new and stimulating possibilities of pharmacological strategies directed against the BBB disruption, albumin pass to brain extracellular space, astrocyte activation, DNA synthesis, and so on.

## Abbreviations

Alb: Albumin
BBB: Blood-brain barrier
BPA: Bovine plasma albumin
BrdU: Bromodeoxyuridine
DNA: Deoxyribonucleic acid
ECoG: Electrocorticography
EEG: Electroencephalography
FBS: Fetal bovine serum
FO: Foramen ovale
GFAP: Glial fibrillary acidic protein
HEPES: N-2-Hydroxyethylpiperazine-N-2-ethanesulfonic acid
HPA: Human plasma albumin
IP_3_: Inositol 1,4,5-trisphosphate
Kir: Potassium inward-rectifier conductance/channel
MRi: Magnetic resonance imaging
PBS: Phosphate buffered saline
p-Alb: Plasma albumin
s-Alb: Serum albumin
SPECT: Single photon emission computer tomography
TGF: Transforming growth factor
TLE: Temporal lobe epilepsy
v-EEG: Video-electroencephalography.
